# Analysis of professional implication as a tool of permanent education in health*

**DOI:** 10.1590/1518-8345.3114.3189

**Published:** 2019-10-07

**Authors:** Flávio Adriano Borges, Cinira Magali Fortuna, Adriana Barbieri Feliciano, Márcia Niituma Ogata, Maristel Kasper, Mônica Vilchez da Silva

**Affiliations:** 1 Universidade Federal de São Carlos, Departamento de Enfermagem, São Carlos, SP, Brazil.; 2 Scholarship holder at the Coordenação de Aperfeiçoamento de Pessoal de Nível Superior (CAPES), Brazil.; 3 Universidade de São Paulo, Escola de Enfermagem de Ribeirão Preto, PAHO/WHO Collaborating Centre for Nursing Research Development, Ribeirão Preto, SP, Brazil.; 4 Scholarship holder at Fundação de Amparo à Pesquisa do Estado de São Paulo (FAPESP), Brazil.; 5 Departamento Regional de Saúde, Centro de Desenvolvimento e Qualificação para o Sistema Único de Saúde, Araraquara, SP, Brazil.

**Keywords:** Nursing, Public Health Nursing, Public Health, Continuing Education, Health Policy, Qualitative Research, Enfermagem, Enfermagem em Saúde Pública, Saúde Pública, Educação Permanente, Políticas de Saúde, Pesquisa Qualitativa, Enfermería, Enfermería em Salud Publica, Salud Publica, Educación Permanente, Política de Salud, Investigación Cualitativa

## Abstract

**Objective:**

analyze professional implication with the support of humanization and articulators of permanent education in health as a tool of Permanent Education in Health.

**Method:**

this is an interventional study of qualitative approach, based on the theoretical reference of Institutional Analysis. Thirty-five humanization supporters and/or permanent education articulators participated in this study. Semi-structured interviews, monthly meetings, restitution meetings and a daily logbook were used as tools for data production. The material was analyzed according to the principles of the study reference and the results were presented according to the ideological, organizational and libidinal dimensions of professional implication.

**Results:**

this study identified a contradiction when finding a professional profile for the development of support and articulation; feelings of discouragement, pessimism and optimism in the development of these roles; influences of the nursing profession on the development of support and articulation; length of professional service; and the absence/presence of desire in such development.

**Conclusion:**

the analysis of professional implication consisted of a powerful tool generating training processes. It allowed learning and reflection of the practice through analysis of the actions performed by the professionals, generating changes in the conception of work in health.

## Abstract (pt)

**Objetivo:**

analisar a implicação profissional com os apoiadores de humanização e os articuladores de educação permanente em saúde como um dispositivo de Educação Permanente em Saúde.

**Método:**

pesquisa-intervenção, de abordagem qualitativa, fundamentada no referencial teórico da Análise Institucional. Participaram da pesquisa 35 apoiadores de humanização e/ou articuladores de educação permanente. Foram utilizadas entrevistas semiestruturadas, encontros mensais, encontros de restituição e o diário de pesquisa como instrumentos para a produção dos dados. A análise do material foi feita segundo os princípios do referencial do estudo e os resultados foram apresentados conforme as dimensões ideológica, organizacional e libidinal da implicação profissional.

**Resultados:**

foram identificadas: a contradição em se pensar em um perfil profissional para o desenvolvimento do apoio e da articulação; os sentimentos de desânimo, pessimismo e otimismo no desenvolvimento dessas funções; os atravessamentos que a profissão de enfermagem exerce no fazer apoio e articulação; o tempo de exercício profissional; e a ausência/presença do desejo no desenvolvimento das mesmas.

**Conclusão:**

a análise de implicação profissional consistiu em um potente dispositivo gerador de processos formativos. Ela possibilitou o aprendizado e a reflexão da prática por meio da análise das ações executadas pelos profissionais, gerando transformações da concepção do trabalho em saúde.

## Abstract (es)

**Objetivo:**

analizar la implicación profesional con los colaboradores de humanización y los articuladores de educación permanente en salud como un dispositivo de Educación Permanente en Salud.

**Método:**

investigación-intervención, de abordaje cualitativo, fundamentado en el referencial teórico del Análisis Institucional. Participaron de la investigación 35 colaboradores de humanización y/o articuladores de educación permanente. Fueron realizadas encuestas semiestructuradas, encuentros mensuales, encuentros de restitución y el diario de investigación como instrumentos para la producción de los datos. El análisis del material fue hecho según los principios del referencial del estudio y los resultados fueron presentados según las dimensiones ideológica, organizacional y libidinal de la implicación profesional.

**Resultados:**

fueron identificadas: la contradicción en pensarse en un perfil profesional para el desarrollo del apoyo y de la articulación; los sentimientos de desánimo, pesimismo y optimismo en el desarrollo de esas funciones; los atravesamientos que la profesión de enfermería ejerce en el hacer apoyo y articulación; el tiempo de ejercicio profesional; y la ausencia/presencia del deseo en el desarrollo de las mismas.

**Conclusion:**

el análisis de implicación profesional consistió en un potente dispositivo generador de procesos formativos. Ella posibilitó el aprendizaje y la reflexión de la práctica por medio del análisis de las acciones que fueron ejecutadas por los profesionales, generando transformaciones de la concepción del trabajo en salud.

## Introduction

Permanent Education in Health (EPS) has been addressed in several scientific studies developed by nurses associated with the training of health professionals for technical improvements of their daily practices^(^ 1 ^-^ 2 ^)^. However, the National Policy for Permanent Education in Health (PNEPS) has an approach that goes beyond the character of punctual training, looking more like a process aiming to place daily work actions in a collective analysis to allow changes in practices and training. This is due to the understanding that the problems in the territories and those experienced in the daily work in health have a complex nature, requiring solutions based on the context of the professionals inserted in that reality and knowledge that goes beyond the technical fields of health professions^(^ 3 ^)^.

Because its training characteristic is based on constructions of collective interprofessional proposals contextualized according to reality, the EPS has a strong transforming power - when used in this perspective - to favor dialogue, cope with conflicts, develop teamwork, expand the ability to analyze work processes and the daily reality^(^ 4 ^-^ 6 ^)^.

Nursing has performed educational actions in all its professional dimensions due to its historical trajectory of attention and management of work and, consequently, it conducts improvements and training of health professionals, aiming to enhance quality and improve care provided to the population. Then, it has recognized the need to become increasingly closer to training through health work^(^ 7 ^)^.

At the same time, in addition to the lack of appreciation and attention, all health professionals are distant from moments dedicated to reflect on work processes in their respective scenarios^(^ 8 ^-^ 9 ^)^. On the other hand, when these moments actually happen, a model focused on the transmission of vertical knowledge still prevails, from those who ‘know more’ to those who ‘know less’^(^ 10 ^)^. Also, a great challenge has to be addressed in terms of changing the formats and conception of health worker education, becoming more critical and emancipatory.

Studies indicate obstacles in understanding EPS and creating spaces for reflect on work processes, and the use of training strategies that consider the context experienced by health professionals, discussing their practices^(^ 1 ^-^ 5 ^)^. The number of scientific studies is still insufficient to indicate effective tools to produce reflection through the context experienced by health professionals and that comply with the EPS principles.

In this path toward changes, diversification of methodological proposals focused on discussing learning approaches allowing health professionals to find solutions for their daily problems and develop critical and reflective thinking about the reality, directly contributes to a more resolute health system of superior quality^(^ 7 ^)^.

In dialogue with problematization and as a concept resulting from the theoretical reference of an Institutional Analysis, the analysis of professional implication can act as a powerful tool for the development of EPS. It is based on a concept, initially proposed by René Lourau in the field of social analysis^(^ 11 ^)^, and later developed by Gilles Monceau as one of the eight principles of *socio-clinique institutionnelle*, which corresponds to one of the intervention categories of Institutional Analysis and which has been developed since 2000s^(^ 12 ^)^.

The implication for this reference does not involve engagement or commitment of participants in the development of their practices, but the way through which they relate to institutions^(^ 11 ^-^ 14 ^)^. It can take place at the ideological, libidinal and organizational level^(^ 11 ^)^.

Institutions, in turn, do not refer to equipment or buildings (considered in this reference as organizations), but to an immaterial dimension related to standards and rules socially built and established, such as family, education, health, among others^(^ 11 ^)^.

The principles of *socio-clinique institutionnelle* do not consist of a list of rules to be observed, but characteristics that guide institutional interventions. These principles are: analysis of orders and demands; participation of individuals in the tool; work of analyzers; analysis of transformations that occur as the work progresses; application of restitution categories; intention of knowledge production; attention to institutional contexts and interferences; and, as explained above, analysis of implications^(^ 12 ^)^.

Therefore, analyzing the professional implication means analyzing the relationship the individuals establish with their profession (identified as an institution) and with the other institutions where they act^(^ 12 ^)^.

The interest in exploring the study theme results from a previous study conducted in six municipalities in the State of São Paulo*, which indicate the absence of meaning when performing a supporting role, identified by the supporters themselves and supported professional teams. Then, it justifies the selection of professional implication analysis as a tool for reflection on EPS support and articulation roles.

Based on these concepts, the implementation of social clinical interventions in the field of nursing and health is an innovative strategy as it reveals how institutions operate in professional practices^(^ 15 ^)^, supporting the achievement of goals set by the EPS. Then, this study aimed to analyze professional implications with EPS humanization supporters and articulators as an EPS tool.

## Method

This is an interventional study^(^ 16 ^)^ of qualitative approach, based on the theoretical and methodological reference of Institutional Analysis^(^ 11 ^)^ in its institutional social clinical principle^(^ 12 ^)^.

Data presented in this study are part of a doctoral thesis developed in articulation with projects to strengthen the Brazilian Health System (SUS), such as: Research for SUS* and the INOVASUS Award**.

This study was developed with supporters of the Humanization Policy and articulators of Permanent Education in Health from all 24 municipalities that comprise a Regional Health Department (DRS) in the State of São Paulo. According to data of 2015 from the Brazilian Institute of Geography and Statistics, this region has 991,129 inhabitants, and 16.6% of the municipalities present a Human Development Index (HDI) above 0.8, which corresponds to a small number of municipalities with a high HDI.

The inclusion criterion was EPS humanization supporter and/or articulator in any of the municipalities belonging to this DRS. The exclusion criterion was professionals on leave or away from their role of EPS support and articulation.

The participants were invited in connection with the Development and Qualification Center for SUS (CDQ-SUS) of the DRS in question, as it has monitored the actions conducted by EPS humanization supporters and articulators since 2007. Also, this CDQ-SUS had an active participation in the creation of this role focused on the articulation and implementation of the National Policies of Humanization and EPS in the municipalities that comprise this DRS. In general, the same individuals are invited by the municipal health administrator to hold this role in the DRS.

This study and its general objective were explained to the participants in line with the presentation of an informed consent form (ICF), and no supporter or articulator refused to participate; they signed the ICF and received a copy of the ICF signed by the researcher in charge.

This study had the participation of 35 health professionals who had a support and/or articulation role in their respective municipalities. It should be noted that, in some municipalities, one professional may hold both roles. It happens especially in smaller municipalities, where the number of health professionals is low.

Data were collected for this study through: a) semi-structured interviews conducted between August and November 2016, which had the following study questions: 1 - Tell me what you have developed as an EPS humanization supporter and articulator in your municipality; 2 - Tell me the enablers and challenges for the development of support in your municipality; 3 - Tell me what you understand by EPS support and articulation; b) 10 monthly meetings with EPS supporters and articulators, held from September 2016 to June 2017, for the development of EPS with them and creation of strategies and possible answers to the questions that involved their work in the territory, reflecting and analyzing their professional practices; c) 2 meetings held in August and September 2017 for study data restitution; d) daily logbook of the first author.

All meetings and interviews were previously scheduled with the participants, recorded in MP3 format and fully transcribed, seeking to ensure participant privacy and freedom of speech. Monthly and restitution meetings were held in the city hosting this DRS, with an average duration of 3 hours each, and the interviews in the respective municipalities of the supporters and articulators lasted 35 minutes, on average.

The transcriptions were prepared for analysis in three steps: 1) the transcription work itself, where a scene or a testimony is transferred to the written form; 2) the transposition work, when words and gestures are reconsidered and restituted through the researcher’s words; and 3) the reconstruction work that consists of the narrative discussed around the main categories of the analysis^(^ 17 ^)^.

The transcriptions of the meetings and interviews were submitted to a process of re-reading for possible correction and removal of sections, words and names that could identify the participants, and for speech analysis. Then, the transposition work was performed, when the transcriptions were read several times, adding considerations written in the logbook which were related to non-verbal readings and expressions identified in the speech (pauses, moments of tension, easy talk, laughter , irony, etc.). And finally, the reconstitution work was conducted to analyze the material transcribed and transposed according to the institutional social clinical principles, looking for fragments, contexts and expressions that would somehow indicate the professional implications of the individuals in the ideological, organizational and libidinal dimensions.

After analyzing the transcribed material and the notes from the logbook, the restitution process started with the presentation of some analyses of professional implications of the supporters and articulators, previously conducted with the material of the interviews, the monthly meetings and the logbook. Then, the process of collective analysis of these data started. That is, after sharing a pre-analysis of the material, it was possible to trigger new analytical processes with the study participants, enriching data production and conducting the intervention proposed in this study.

The results obtained with the different data production tools were organized considering the ideological, organizational and libidinal dimensions of professional implication of EPS supporters and articulators. The study results and developments were explained, showing the effects of the professional implication analysis as a tool triggering training processes.

This study was approved by the Research Ethics Committee of the Nursing School of Ribeirão Preto, at Universidade de São Paulo, protocol nº 1.568.447, ensuring confidentiality of all data produced. Then, the excerpts of participant speeches are identified in this study by the roles followed by a cardinal number (for example, articulator 01, supporter 02, supporter/articulator 03, etc.), and the notes from the logbook will be presented with their corresponding dates.

## Results

This study had the participation of 35 supporters and/or articulators: 16 nurses, 6 psychologists, 3 occupational therapists, 3 physical therapists, 2 nutritionists, 1 administrator, 1 speech therapist, 1 psychopedagogue, 1 odontologist, and 1 pharmacist.

The ideological dimension of professional implication refers to what the individuals believe. In this case, it consists in the ideological relationship supporters and articulators establish with the support and articulation of EPS. Then, some questions pointed to this dimension, among them the existence (or not) of a profile for performing their roles, included in several instruments used in this research.

In the process of restitution and collective analysis of data, two main ideas of professional profile emerged. One was related to commitment, involvement and interest of the supporter and articulator to perform the support/articulation work, and one was a skill for the development of these two roles; at first, this skill seemed an innate characteristic of the professional (some of them had it, others did not). *I see a profile more in this issue of commitment* (...). *One had an interest and ended up prioritizing other things* (...). *So, it mixes profile, interest, commitment...* (articulator 10); *I think a little beyond that, this profile, for me, has more to do with the skills to perform this role, which are natural to each human being, each professional* (supporter 13).

After the process of collective analysis of data and discussions, supporters and articulators concluded there is not an exact profile for the development of their respective roles. This idea emerged as an exercise of a role that could be built, produced, developed through professional and life experiences. It is something learned while performing the roles. *I was wondering if there is a supporter and articulator profile. Is it desirable for this person to have this, this, and that?* (...) *I don’t know if I see anything that is predetermined, but I think, much more, about what makes a person remain in this role* (supporter 02); *I remember when I was invited to be an EPS articulator* (...) *this profile issue has always bothered me. Why? How did they see that I have this profile if I didn’t see it? What profile is it? So today I understood that it can be built* (articulator 13).

The organizational dimension of professional involvement refers to the material and organizational basis (as the name suggests) the individual establishes with the profession. At data restitution, that is, when the analysis of implication may become more evident, a table was presented showing professions of supporters and articulators of that DRS. At that moment, the questions related to the professional practice of the nurse were explained, since 47% (16 of the 35 participants) were nurses. The influence of nursing was present in the testimony of interviewees*. And thinking a little more about this analysis of implication* (...)*, I always think of something, which is the fact of being a nurse* (supporter 03); *It’s something I like, but sometimes I felt frustrated* (...)*. Because I couldn’t do the EPS activities and stop. For example, if we are in the meeting and have an emergency, I, as a nurse, would have to stop and help in the emergency room* (supporter/articulator 01); *So care nurses are the good nurses of the unit. I’d like to be a care nurse, the good nurse* (...) *driving reflections generates conflict* (articulator 03).

In the perspective of transformations caused by the social clinical intervention, a supporter provides some clues to explain why nurses are the right persons for these roles, managing and organizing the health work while developing them*. I understand the reason for that* (...) *nursing has some aspects involving administration. We study administration at the university. Then, I think: Why did I work in that hospital laundry when I was an intern? I had to understand the fabric, the product... what another professional will visit a hospital laundry?* (...) *then we have that on our back* (supporter 10).

Time was another issue related to the organizational dimension of professional implication, identified in the speeches of participants in the collective analysis of the restitution sessions. *I keep thinking that when you are in the care role, it becomes difficult. Then, a pregnant woman arrives seeking care, will you leave the pregnant woman there to hold a meeting? We feel divided* (refering to the meeting as a support activity) (supporter 01). *It is interesting to note that the lack of time for the development of support and articulation is always present in the speeches and interviews* (logbook, Nov. 18, 2016).

Through the data restitution process, evidence regarding the poor use of time was identified and exposed by the participants. *I don’t see myself exhausted from 7 am to 4 pm, from Monday to Friday* (...)*. There are peak times in the unit and times when things calm down... it happens every day. I’ve already worked in four units, in very large and very small cities, I’ve worked in rural units and urban units... and it’s always or more or less the same routine* (articulator 04).

Regarding the libidinal dimension, which is related to the desire and affection of EPS supporters and articulators while performing their respective support and articulation roles, issues were observed that express the presence and absence of desire for the development of EPS support and articulation roles. *I don’t know if I’m the right person for this role* (...) *Sometimes I get very discouraged, you know? I don’t know if it’s lack of motivation or dedication* (supporter/articulator 06). *What happens is that we are losing the feeling, the will* (...) *Sometimes I don’t recognize myself anymore. It’s strange* (supporter 07); *And since basic attention has always been ‘the apple of my eye,’ I didn’t think twice to accept, so I stayed here* (...) *I love these policies* (referring to the National Humanization Policy and the National EPS Policy) (supporter/articulator 07); *I’ve always enjoyed working here, in this municipality. I even have another job and I told them I was going to leave here three months ago, I was going to keep the other job, but I couldn’t leave it here* (articulator 01).

Another issue related to the libidinal dimension of professional involvement was the expression of discouragement and pessimism while performing EPS support and articulation roles. Such feelings indicate the absence of a power to leverage the support and articulation roles. *So what keeps me here? The meaning? I don’t know. I don’t know anymore. To tell you the truth, I can’t stand it anymore* (head down, expressing discouragement) (supporter 10); *Wow! My feeling is that the supporters and articulators showed discouragement at today’s meeting. I can’t understand very well whether their silence has to do with the political moment of the municipality or a result of the meeting and reflections generated by the meeting itself* (logbook, September 29, 2016).

These feelings are linked with the idea of feeling overloaded and, therefore, they find it difficult to develop actions that reflect quantitative data in the indexes presented by the municipalities; a sense of not being able to objectively demonstrate the work conducted as a supporter and articulator. *You keep doing it, you can’t say no, but you can’t do it completely, you always start, but you don’t finish it, things start to accumulate and you are overloaded, then you are pressured, you put pressure on yourself and then feel discouraged* (articulator 11); *As she said, logic is focused on the procedure. Then I can’t measure or show my work. Numbers. And the EPS doesn’t work in this perspective* (supporter 05).

After discussing data in a restitution meeting, based on the work performed by some articulators and supporters in the EPS perspective, a transformation was observed regarding the logic of EPS support and articulation production, understanding that they are not only numerical data supporting the indices obtained by the municipality in question, but also a tool that produces qualitative improvement in the work provided to the population in general. *You can’t measure the work* (...) *you will only know whether or not that unit has progressed in the work process if they see that their work has not favored, for example, new demands received by the unit* (supporter/articulator 03); *I think that our actions should be based on our reality. For example: this job has to be ready, 100%, but it’s not the reality* (...) *you have to be optimistic while considering the reality. Is it possible to achieve?* (articulator 07).

This dimension of professional implication, especially regarding the absence of desire for the development of roles, was gradually transformed during the intervention process of this study. This fact could be confirmed as an indicator, for example, of reduced absenteeism in the monthly meetings and the explicit demand of participants for a formal training process (course) in EPS support and articulation.

## Discussion

Regarding the ideological dimension presented by the participants in the development of this study, some authors^(^ 13 ^,^ 18 ^-^ 21 ^)^, who also conducted studies with supporters, found questions related to the profile for the development of this role. They reported that the developments of the supporter practice also depend on mobilization and commitment of the subjects to the transformation of work processes in health^(^ 22 ^)^.

A social clinical intervention and the analysis of professional implication allowed reflections on the functioning of these roles of EPS support and articulation with the professionals that perform them in their daily lives. According to data produced in this study, some profiles have to be achieved or profiles are required for the roles. For this reason, courses are prescribed and skills are determined. The point highlighted and reiterated in this study is that support and articulation are performed through an encounter, a mediator, where one learns from performing a role^(^ 23 ^-^ 24 ^)^, that is, the role is learned from daily experience^(^ 3 ^)^ and, above all, from analyzing what, how, what for, and why the work is conducted.

Regarding the organizational dimension of professional implications, this study identified contributions and interferences of nursing while supporting and articulating EPS. This finding put the ‘nursing profession’ in evidence. This profession, which carries a social image of leadership, control, technical rigor, systematization and planning, was analyzed with the participants in a restitution meeting. Surrounded by discomforts when they realized how much they carry from their professional training while developing their roles of EPS support and articulation, it was possible to perceive a transformation in participant learning, as they started to identify characteristics of the profession itself, associated with the development of these roles. This fact shows that an implication analysis also produces knowledge^(^ 16 ^)^.

Another characteristic that emerged in the organizational dimension of professional implications was the time devoted to the development of EPS support and articulation roles. Other studies have also identified that short time, justified by a hard work routine of health professionals, represents one of the challenges for the development of EPS articulation^(^ 25 ^)^ and the professional practice of nurses^(^ 26 ^-^ 27 ^)^.

Some authors^(^ 23 ^)^ advocate the allocation of a specific workload to EPS support and articulation within the hours worked by professionals in direct health care (nurses, psychologists, occupational therapists, etc.). They also recommend the adoption of strategies, such as the implementation of directives or laws seeking legal formalization of this distribution of workload, ensuring formal time to those who perform both roles. Measures like these attempt to ensure the implementation of these roles, providing a legitimate space and recognition by other health workers and municipal administration staff. However, defining a specific workload for the development of these roles does not eliminate the need for a constant analysis of the work developed, since, while this action seems to be strong, it may lose its creative, inventive and effective contribution to the development of EPS support and articulation as it becomes an institution.

Regarding the libidinal dimension of professional implications in the development of EPS support and articulation, 100% of the supporters and articulators from the studied contexts were appointed by the municipal administrator to their respective roles. In addition, many of them were not asked whether they would like to hold such roles, which affects their performance in such functions.

Another study conducted in a large municipality in the country side of the State of São Paulo reported 83% of local supporters has been invited by the administration to assume this role, also showing discouragement and some immobility among the supporters^(^ 28 ^)^. However, a study conducted in another municipality in the same state reported desire regarding the role of supporters, concluding that the workgroups of that location were enthusiastic about the development of this role^(^ 24 ^)^.

The feeling of discouragement and pessimism in EPS support and articulation, also observed in the libidinal dimension of professional implications, permeate the process of daily search for legitimacy and recognition as supporters and articulators through visibility of the work performed. These two roles are important management tools, but they have found several obstacles, such as teamwork challenges, improper working conditions, insufficient investment in health, and structural and managerial issues in health^(^ 23 ^)^.

This is also due to the capture of the driving force (the productive feeling provided by the encounter), of consolidated forces that resist the emergence of new styles, that can be powerful or not, of performing EPS support and articulation^(^ 29 ^)^. In the institutional analysis, the driving forces that generate movement would be the institution forces, and the forces that tend to prevent changes are the instituted forces^(^ 11 ^)^.

The attempt to set a goal and quantify the actions of EPS support and articulation seems to be a reproduction of consolidated work, replacing the active worker with an appreciation of inactive work, which in this case would correspond to the mere quantification of the work provided^(^ 30 ^)^. Then, it is possible to produce qualitative and quantitative indicators based on support and articulation actions, since the progress and positive results are observed in the daily life of health work^(^ 3 ^)^.

Whether or not desire is present in the development of EPS support and articulation roles, it is important to analyze it, since it comprises one of the dimensions of professional implications which somehow will help achieve a transformation in the work process.

Then, the innovative characteristic of this study consists in showing that professional implication analysis can be used as a powerful tool for the development of EPS. It reinforces the insufficient number of studies addressing effective strategies to trigger reflections on the context experienced by health professionals, according to the PNEPS principles. However, it presents the limitation of not addressing the transformations perceived in the daily work performed by these health professionals. Then, the development of studies analyzing the professional practice of these individuals longitudinally can show the changes in the daily work in different realities.

## Conclusion

A professional implication analysis with EPS humanization supporters and articulators was of a powerful tool for the generation of reflection and training processes. From individual experiences, challenges and potentialities were recognized, which are related to these roles but not yet legitimized in health. This study also identified changes in participant learning, in the daily work of supporters and articulators, generating transformations in the concept of EPS support and articulation.

## Footnotes

* Study funded by the Ministry of Health, the National Council for Scientific and Technological Development (CNPq), the Health Department of the State of São Paulo, and the Foundation for Research Support of the State of São Paulo (FAPESP). This study was awarded by the Pan American Health Organization and the Ministry of Health (Inovasus Award).

* Study funded by a partnership involving the Ministry of Health, the National Council for Scientific and Technological Development (CNPq), and Foundation for Research Support of the State of São Paulo (FAPESP).

** Innovation Award for the Brazilian Health System (INOVASUS) (INOVASUS Education Management in Health), an initiative of the Ministry of Health in partnership with the Pan American Health Organization.
